# Identification of a pyroptosis-related prognostic signature in breast cancer

**DOI:** 10.1186/s12885-022-09526-z

**Published:** 2022-04-20

**Authors:** Hanghang Chen, Haihua Luo, Jieyan Wang, Jinming Li, Yong Jiang

**Affiliations:** 1 Guangdong Provincial Key Laboratory of Proteomics, State Key Laboratory of Organ Failure Research, Department of Pathophysiology, School of Basic Medical Sciences, Southern Medical University, No.1023 Shatai South Road, Guangzhou, 510515 Guangdong Province China; 2 Department of Bioinformatics, School of Basic Medical Sciences, Southern Medical University, Guangzhou, 510515 China

**Keywords:** Pyroptosis, Breast Cancer, Prognosis, Tumor immune microenvironment, Tumor mutational burden

## Abstract

**Background:**

The relationship between pyroptosis and cancer is complex. It is controversial that whether pyroptosis represses or promotes tumor development. This study aimed to explore prognostic molecular characteristics to predict the prognosis of breast cancer (BRCA) based on a comprehensive analysis of pyroptosis-related gene expression data.

**Methods:**

RNA-sequcing data of BRCA were collected from The Cancer Genome Atlas (TCGA) and Gene Expression Ominibus (GEO) datasets. First, pyroptosis-related differentially expressed genes (DEGs) between normal and tumor tissues were identified from the TCGA database. Based on the DEGs, 1053 BRCA patients were divided into two clusters. Second, DEGs between the two clusters were used to construct a signature by a least absolute shrinkage and selection operator (LASSO) Cox regression model, and the GEO cohort was used to validate the signature. Various statistical methods were applied to assess this gene signature. Finally, Single-sample gene set enrichment analysis (ssGSEA) was employed to compare the enrichment scores of 16 types of immune cells and 13 immune-related pathways between the low- and high-risk groups. We calculated the tumor mutational burden (TMB) of TCGA cohort and evaluated the correlations between the TMB and riskscores of the TCGA cohort. We also compared the TMB between the low- and high-risk groups.

**Results:**

A total of 39 pyroptosis-related DEGs were identified from the TCGA-breast cancer dataset. A prognostic signature comprising 16 genes in the two clusters of DEGs was developed to divide patients into high-risk and low-risk groups, and its prognostic performance was excellent in two independent patient cohorts. The high-risk group generally had lower levels of immune cell infiltration and lower activity of immune pathway activity than did the low-risk group, and different risk groups revealed different proportions of immune subtypes. The TMB is higher in high-risk group compared with low-risk group. OS of low-TMB group is better than that of high-TMB group.

**Conclusion:**

A 16-gene signature comprising pyroptosis-related genes was constructed to assess the prognosis of breast cancer patients and its prognostic performance was excellent in two independent patient cohorts. The signature was found closely associated with the tumor immune microenvironment and the potential correlation could provide some clues for further studies. The signature was also correlated with TMB and the mechanisms are still warranted.

**Supplementary Information:**

The online version contains supplementary material available at 10.1186/s12885-022-09526-z.

## Background

Breast cancer (BRCA) is a heterogeneous disease with a high level of morbidity, accounting for 30% of cancer diagnoses in females in 2020 [1]. Currently, treatment strategies of for BRCA mainly consist of surgery, chemotherapy, endocrine therapy, trastuzumab-based antibody therapy and radiation therapy on the basis of disease stage and pathological characteristics [2]. Despite the dramatic improvement in breast cancer prognosis over the previous decades, innovative methods are still needed to identify high-risk patients. Moreover, treatment plans should be individualized due to the heterogeneity of BRCA. The most significant advance in the characterization of cancer heterogeneity over the past few decades may be the application of DNA microarray [3] and next-generation sequencing [4] technologies over the past few decades. In addition to clinicopathological features, individual gene signatures could provide alternative information to predict breast cancer prognosis [5].

Pyroptosis is a recently discovered type of programmed cell death that can lead to cell swelling and cell membrane rupture and trigger a strong inflammatory response related to innate immunity [6]. Pyroptosis plays a dual antitumor and tumor-promoting role in the occurrence and development of tumors. On the one hand, it could cause local inflammation and subsequently provide an opportunity to relieve immunosuppression of the tumor microenvironments (TME) [7]. Additionally, chemotherapy drugs can trigger tumor cell pyroptosis through different mediators [8]. On the other hand, excessive inflammatory mediators released during pyroptosis are tightly related to the tumorigenesis [9], drug side effects [10, 11], resistance to chemotherapeutics [12] and the acceleration of tumor development in different cancers [13].

The TME plays complex and paradoxical roles in cancers, which elicit both beneficial and adverse consequences for tumorigenesis [14]. A variety of immunotherapies, such as immune checkpoint blockade, have been used in the treatment of cancer and yielded satisfactory response rates [15]. However, a highly immunosuppressive TME accelerates tumor progression [16]. Increasing evidence shows show that in the TME the immune cells contribute to tumor metastasis [17]. To date, the specific relationship between pyroptosis and the TME as well as their roles in BRCA progression are still unclear.

In the present study, we aimed to construct a scoring model based on pyroptosis-related genes to predict the prognosis of BRCA patients. First, we classified 1053 female BRCA patients from the TCGA dataset into two clusters according to their expression profiles of the pyroptosis-related genes. Second, DEGs between the two clusters were utilized to construct a pyroptosis-related signature by the LASSO-Cox method. Finally, the signature was validated via multiple approaches. The signature could predict the prognosis of BRCA patients and indicate immune infiltration. Our findings suggest a potential connection between pyroptosis, prognosis and the tumor microenvironment of BRCA patients, which has seldom been reported earlier to date.

## Methods

### Datasets

The RNA-seq and mutation data of female BRCA patients and the corresponding clinical data were downloaded from the TCGA data portal (https://portal.gdc.cancer.gov/repository). The 1164 samples included 111 normal tissues and 1053 tumor tissues. When we performed conjoint analyses, the samples with missing data were deleted. 900 patients survived while 142 patients had passed away at the time of the last follow-up.

In addition, the breast cancer RNA expression data with paired clinical and follow-up information of four external validation cohorts (including 636 samples, GSE20685+ GSE20711+ GSE42568+ GSE88770) [18–21] were downloaded from the GEO database (https://www.ncbi.nlm.nih.gov/geo/). We merged the four validation cohorts and removed the batch effect. We also adjusted and normalized the RNA expression data of the two datasets with the “limma” (version 3.49.4) and “sva” (version 3.42.0) R packages. 465 patients survived while 171 patients had passed away at the time of the last follow-up.

### Identification of differentially expressed pyroptosis-related genes

We identified 52 pyroptosis-related genes from prior reviews, and they are presented in Table S1. The “limma” R package was used to identify DEGs between tumor and normal tissues from the TCGA database with a cut-off *p* value of 0.05. The DEGs are annotated as follows: * if *p* < 0.05, ** if *p* < 0.01, and *** if *p* < 0.001. The “pheatmap” R package (version 1.0.12) was used to create a heatmap of the DEGs.

We calculated the TMB of TCGA cohort with mutation data and Varscan software [22] and explored the mutational status of the DEGs in the TCGA cohort.

We divided patients into high- and low-expression groups based on the median expression of 52 pyroptosis-related genes separately. Subsequently we compared the overall survival (OS) between two groups with the “survival” (version 3.2–11) and “survminer” (version 0.4.9) R packages and displayed them with Kaplan–Meier (KM) curves.

### Clustering patients based on the 39 DEGs

By exploring the expression pattern of the 39 pyroptosis-related DEGs in BRCA using the “ConsensusClusterPlus” R package (version 1.57.0), we divided patients from the TCGA cohort into two clusters. We also compared the overall survival between the two clusters.

We calculated and compared the different passways enriched between two clusters with “GSVA” (Gene Set Variation Analysis) package [23] and displayed them in a heatmap.

### Development and validation of the pyroptosis-related gene prognostic signature

We further employed univariate Cox regression analysis (R package “survival”) to evaluate the correlations between the DEGs in different clusters and survival status in the TCGA cohort. The LASSO regression model (R package “glmnet”, version 4.1–2) was then utilized to narrow down the candidate genes and to develop the prognostic model. The risk score was calculated using the following formula: risk score = expression of Gene 1 ∗ β1 + expression of Gene 2 ∗ β2 + … expression of Gene n ∗ βn, where β represents the regression coefficient of the genes in the signature.

The BRCA patients in the TCGA cohort were divided into low- and high-risk groups according to the median risk score, and OS was compared between the two groups via Kaplan–Meier analysis (“survival” and “survminer” R packages). The “survival”, “survminer” and “time-ROC” (version 0.4) R packages were employed to perform 1-year, 3-year, and 5-year ROC curve analyses.

The BRCA patients in the TCGA cohort were divided into stage I-II and stage III-IV cohorts and OS were compared between the two subgroups via Kaplan–Meier analysis separately.

The clinical data (age, stage) of the patients in the TCGA cohort were collected and analysed in combination with the risk score in our independent regression model. Univariate and multivariate Cox regression models were employed for the analysis by the “survival” R package.

### Nomogram construction and calibration

The riskscore and relevant clinical parameters such as age and stage were incorporated into the construction of a prognostic nomogram via “rms” R package (version 6.2–0) to predict 1-, 3-, and 5-year OS of BRCA patients in the TCGA cohort. We used a calibration plot comparing predicted and observed overall survival to evaluate the performance of the prognostic nomogram (method = “boot”, B = 1000). The “time-ROC” (version 0.4) R package was employed to perform 1-year, 3-year, and 5-year ROC curve analysis to evaluate the performance of the nomogram.

We assessed the accuracy of the signature compared with age and stage through the concordance index (c-index) with “pec” R package.

### Display of the heatmap based on the DEGs in the signature and the risk score

A heatmap based on the genes in the signature and the risk score was displayed by the “limma” and “pheatmap” R packages. We also compared the difference in clinical features between the two risk groups and showed them on the heatmap.

### The differences of the mutational status between high- and low-risk groups

We explored the mutational status of the top 20 genes most frequently mutate in the BRCA samples. We further compared the differences between high- and low-risk groups.

### GO and KEGG enrichment analysis of the genes in the signature

BRCA patients in the TCGA cohort were divided into two groups according to the median risk score. The DEGs between the low- and high-risk groups were identified according to the given criteria (|log_2_FC| ≥1 and FDR < 0.05). To further explore the gene functions and pathways between the groups, GO and KEGG analyses were performed by applying the “clusterprofiler” (version 4.1.3) [24], “org. Hs.eg.db” (version 3.13.0) and “ggplot2” R packages.

### Explore the potential of clinical application of our signature

We further compared the enrichment scores of 16 types of immune cells and 13 immune-related signaling pathways between the low- and high-risk groups in the TCGA cohort by employing single-sample gene set enrichment analysis (ssGSEA). The “GSVA” (version 1.41.3) and “GSEABase” (version 1.55.1) R packages were utilized to calculate the scores of infiltrating immune cells and to evaluate the activity of immune-related pathways. The “ggpubr” package was used to display the box plots.

BRCA patients in the TCGA cohort were divided into different immune subtypes: Wound Healing (C1), IFN-gamma Dominant (C2), Inflammatory (C3), Lymphocyte Depleted (C4), Immunologically Quiet (C5), TGF-beta Dominant (C6) [25]. We compared differences of immune subtypes between high- and low-risk groups.

We also summarized documented immune checkpoints and calculated the correlations between the riskscore, expression of every gene in the signature and the expression of immune checkpoints. Subsequently we displayed the correlations with a heatmap.

To further explore the clinical application value of our signature, we calculated the TMB of TCGA cohort with mutation data. Then we evaluated the correlations between the TMB and riskscores of the TCGA cohort. We also compared the TMB between the low- and high-risk groups in the TCGA cohort and displayed that it with “ggplot2” and “ggExtra” R packages.

We calculated the optimal TMB cutoff value using the “surv_cutpoint” function in “survminer” R package and divided the BRCA patients in the TCGA cohort into low- and high-TMB groups. Then we compared OS between the two groups via Kaplan–Meier analysis. In the same way, to evaluate the mixing functions of riskscore and TMB, we divided the BRCA patients into four groups by TMB and riskscore. Then we compared OS among the four groups.

### Statistical analysis

The gene expression between the normal and tumor samples of BRCA was compared via single-factor analysis of variance. The differences of clinical characteristics between high- and low-risk groups were compared with the pearson chi-square test. The differences of immune cells and functions infiltration scores between high- and low-risk groups was compared with wilcox test. The correlation between TMB and riskscore was calculated with spearman rank correlation. The correlations between the riskscore, expression of every gene in the signature and the expression of immune checkpoints were also calculated with spearman rank correlation. All statistical analyses were performed with R software (v4.1.0), and *p* < 0.05 was selected as statistically significant.

## Results

### Identification of 39 DEGs between normal and tumor tissues

**Fig. 1:**
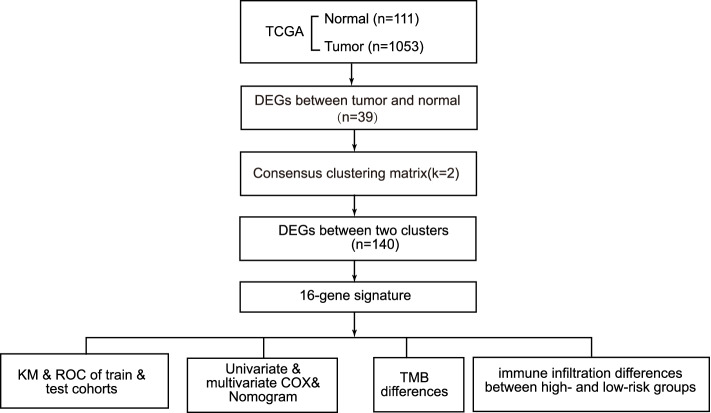
The flowchart of data collection and analyses

The general workflow of this study is displayed in Fig. 1. The 52 pyroptosis-related gene expression levels were compared in 111 normal and 1053 tumor tissues, and we identified 39 differentially expressed genes (DEGs). Among them, 17 genes were downregulated while the other 22 genes were upregulated in the tumor group.

We calculated the OS differences of the 52 genes and listed them in Table S2. We displayed the OS differences of 9 genes closely related with pyroptosis occurrence such as GSDMD, GSDME, CASP8, NLRP7. The expression of the 9 genes were all positively correlated with the OS of BRCA patients.

**Fig. 2:**
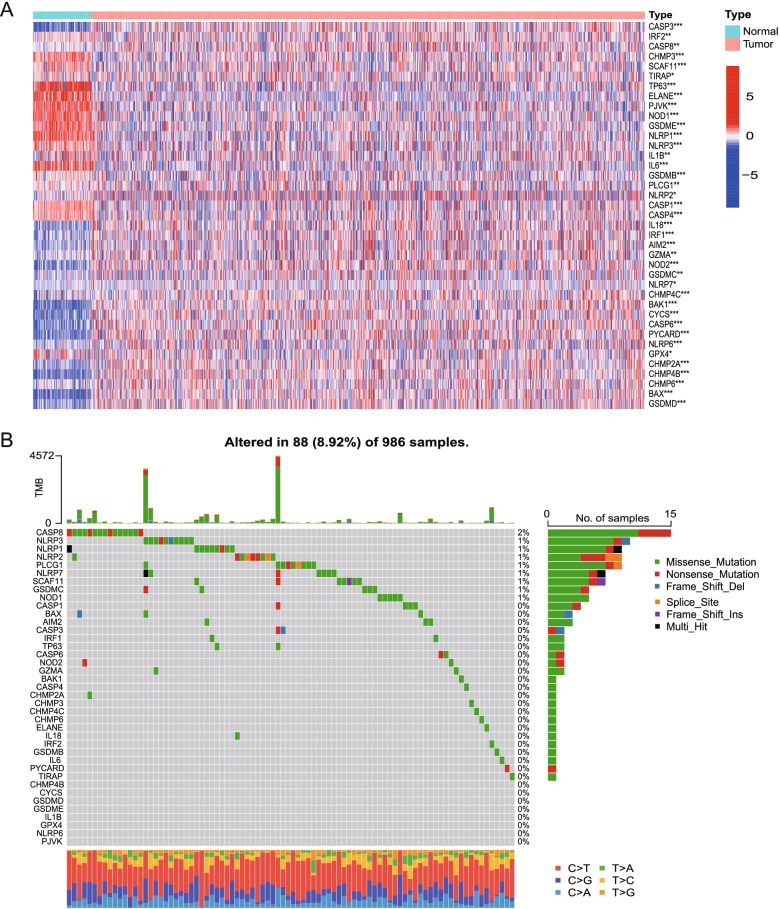
Expressions of the 39 pyroptosis-related DEGs and the interactions among them. **A** The heatmap of the pyroptosis-related differential expressed genes (DEGs) between the normal and the tumor tissues. *P* values were showed as: *p < 0.05, **p < 0.01, ***p < 0.001. **B** The mutational status of every DEG and the TMB of samples. Different colors indicate different mutation types

The RNA expression levels of these DEGs and their differences are presented as a heatmap in Fig. 2A. The mutational status of every DEG are shown in Fig. 2B. The TMB scores of TCGA cohort were listed in Table S3. The most frequently mutant gene is caspase-8 which plays a crucial role in pyroptosis.

### Patients were stratified into two clusters based on the DEGs

**Fig. 3:**
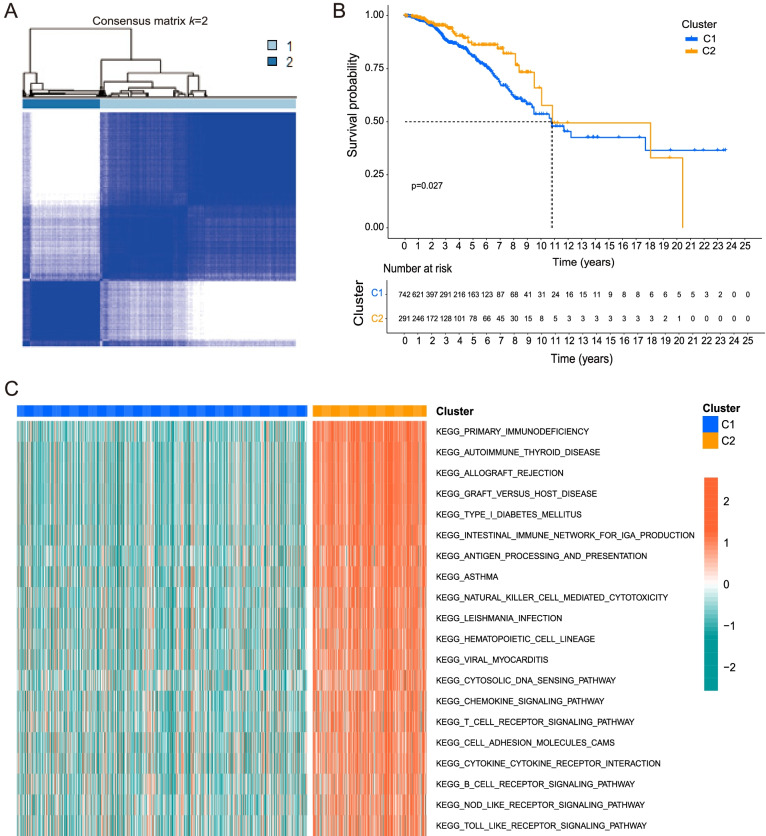
Patients stratified into two clusters based on the DEGs. **A** 1053 BRCA patients were divided into two clusters according to the consensus clustering matrix (k = 2). **B** Kaplan–Meier overall survival (OS) curves of the two clusters. **C** Gene Set Variation Analysis (GSVA) analysis indicated that immune related passways enriched more in C2 than in C1

The consensus clustering analysis indicated that the correlations were high between two clusters when k = 2, which meant that the 1053 BRCA patients could be well divided into two clusters based on the 39 DEGs (Fig. 3A). The overall survival (OS) of cluster C2 was significantly better than C1(*p* = 0.027, Fig. 3B).

GSVA analysis indicated that immune related passways such as primary immunodeficiency passway significantly enriched more in cluster C2 than C1 (Fig. 3C).

### Identification of a 16-gene signature in the TCGA cohort

**Fig. 4:**
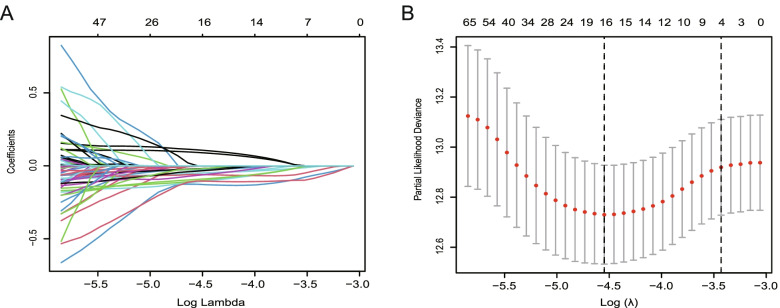
Identification of risk signature in the TCGA cohort. **A** Least absolute shrinkage and selection operator (LASSO) regression of the OS-related genes. **B** Cross-validation for tuning the parameter selection in the LASSO regression

After univariate Cox regression analysis, 140 genes met the criteria of *p* < 0.02 and were retained for further analysis. Among them, 2 genes (ATP8A2, PXDNL) were associated with increased risk (HRs > 1), while the other 138 genes were protective genes (HRs < 1) (Table S4). By performing least absolute shrinkage and selection operator (LASSO) regression analysis, a 16-gene signature was constructed according to the optimum λ value (Fig. 4A, B). The risk score could be calculated using the data in Table 1.

**Table 1:** The genes involved in the signature and their coefficients

No.	Gene name	Coef	No.	Gene name	Coef
1	ATP8A2	0.10922	9	CLIC6	−0.05064
2	PXDNL	0.09159	10	IL27RA	−0.05944
3	IGLL5	−0.00254	11	CXCL1	−0.06532
4	PIGR	−0.02008	12	KLHDC7B	−0.09290
5	LIMD2	−0.02222	13	APOBEC3D	−0.10388
6	PSMB8	−0.02651	14	ELOVL2	−0.10436
7	MATK	−0.03621	15	PLAT	−0.10957
8	CHI3L1	−0.05035	16	KLRB1	−0.12656

### Validation of the risk signature

**Fig. 5:**
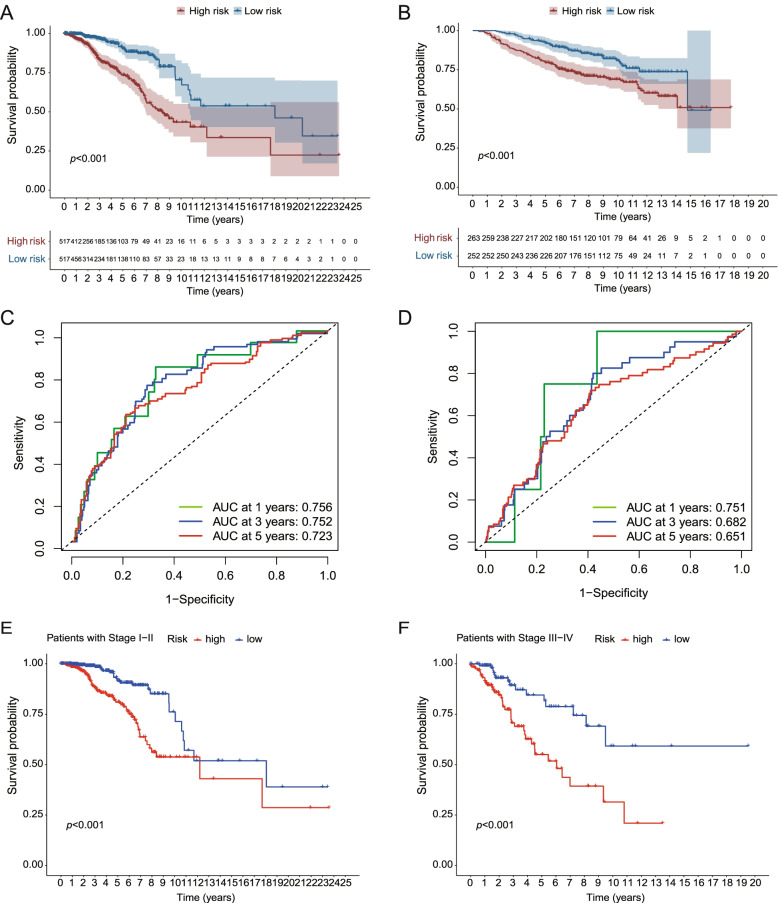
Validation of risk signature in the TCGA and GEO cohorts separately. **A** Kaplan–Meier curves for the OS of patients in the high- and low-risk groups in the TCGA cohort. **B** ROC curves showed the predictive efficiency of the risk scores in the TCGA cohort. **C** Kaplan–Meier curves for the OS of patients in the high- and low-risk groups in the GEO cohort. **D** ROC curves showed the predictive efficiency of the risk scores in the GEO cohort. Kaplan–Meier curves for the OS of low-risk group is better than that of high-risk group in stage I-II (**E**, *p* < 0.001) and stage III-IV (**F**, *p* < 0.001) patients in the TCGA cohort

Patients from the TCGA dataset were stratified into low- and high-risk groups based on the median. A notable difference in OS was detected between the low- and high-risk groups (*p* < 0.001, Fig. 5A). Time-dependent receiver operating characteristic (ROC) analysis was applied to evaluate the sensitivity and specificity of the prognostic model, and the area under the ROC curve (AUC) was separately 0.756 for 1-year survival, 0.752 for 3-year survival, and 0.723 for 5-year survival (Fig. 5C).

Based on the median risk score of the TCGA cohort, 620 patients from the GEO dataset were divided into low- and high-risk groups. OS of low-risk group is also better than that of high-risk group (*p* = 0.001, Fig. 5B). The AUC was separately 0.751 for 1-year survival, 0.682 for 3-year survival, and 0.651 for 5-year survival (Fig. 5D).

Based on the stage, we divided patients into stage I-II and stage III-IV groups. OS of low-risk group is better than high-risk group (both *p* < 0.001, Fig. 5E and F).

**Fig. 6:**
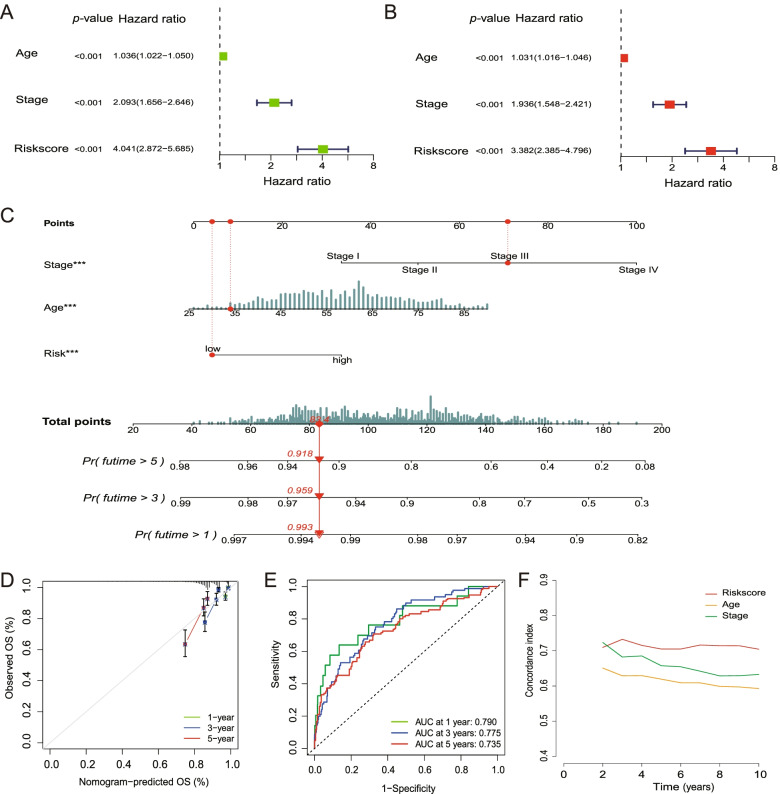
Univariate and multivariate Cox regression analyses and nomogram construction. **A** Univariate analysis for the TCGA cohort. **B** Multivariate analysis for the TCGA cohort. **C** After the multivariate analysis, age, stage, riskscore were significant for the prognosis and selected to construct a nomogram to facilitate the prognosis prediction. The corresponding score of each factor (age, stage and riskscore) was calculated in the nomogram and the total score could be used to predict the OS of BRCA patients. **D** A calibration curve was plotted to indicate the consistency between the actual observed prognosis value and the value predicted by the nomogram. **E** ROC curves showed the predictive efficiency of the nomogram in the TCGA cohort. **F** Compared with age and stage in the prognosis prediction, the signature gave an advantage in C-index

Univariate Cox regression analysis indicated that the riskscore was an independent factor capable of predicting poor survival in the TCGA cohort (Fig. 6A). The multivariate analysis also revealed that, after adjusting for other confounding factors, the riskscore was a prognostic factor (Fig. 6B) for patients.

### Nomogram construction and validation

After the multivariate analysis, age, stage and riskscore were significant for the prognosis and selected to construct a nomogram to facilitate the prognosis prediction (Fig. 6C). The corresponding score of each factor (age, stage and riskscore) was calculated in the nomogram and the total score could be used as a tool for prediction. A calibration curve was plotted to indicate the consistency between the actual observed prognosis value and the value predicted by the nomogram (Fig. 6D).

The performance of the nomogram was assessed and the AUC was separately 0.790 for 1-year survival, 0.775 for 3-year survival, and 0.735 for 5-year survival (Fig. 6E).

Compared with other age and stage in the comprehensive prognosis prediction, the signature gave an advantage in C-index (Fig. 6F).

### Clinical difference between different groups

**Fig. 7:**
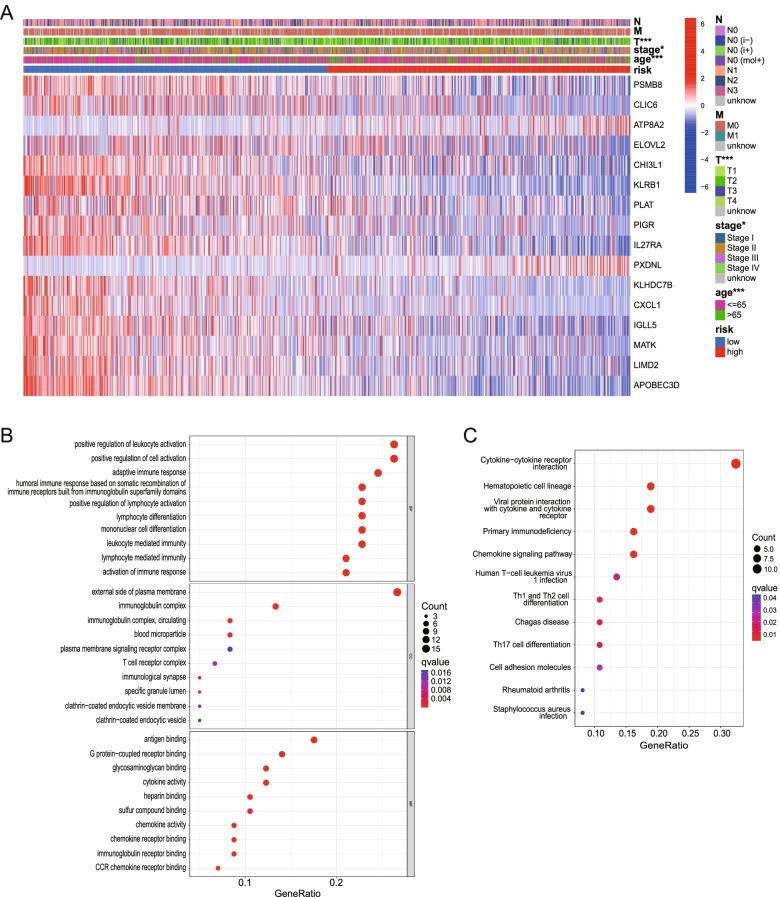
Differences of clinical characteristics and functional analysis. **A** Some clinical characteristics such as age, T are statistically different between high- and low-risk groups. (*p < 0.5, ***p < 0.001). **B** Bubble graph for GO enrichment (q-value: the adjusted *p*-value). **C** Bubble graph for KEGG pathways enrichment analysis

In addition, we plotted a heatmap of the clinical features and genes from the signature for the TCGA cohort (Fig. 7A) and found that the age, T and stage status differed significantly between the low- and high-risk groups (*p < 0.05, ***p < 0.001).

### GO and KEGG mainly indicated immune response

GO enrichment and KEGG pathway analysis of DEGs between different risk groups indicated that the genes were mainly associated with the immune response, chemokine-mediated signaling pathways, and inflammatory cell chemotaxis (Fig. 7B, C).

### The mutational status of different risk groups is generally different

We investigated and displayed the top 20 genes most frequently mutate in the TCGA cohort. The mutational status of high-risk group (Fig. S2A) is generally different from low-risk group (Fig. S2B). For instance, TP53 mutated more frequently in high-risk group whereas CDH1 mutated more frequently in low-risk group.

### The potential clinical application of the risk signature

**Fig. 8:**
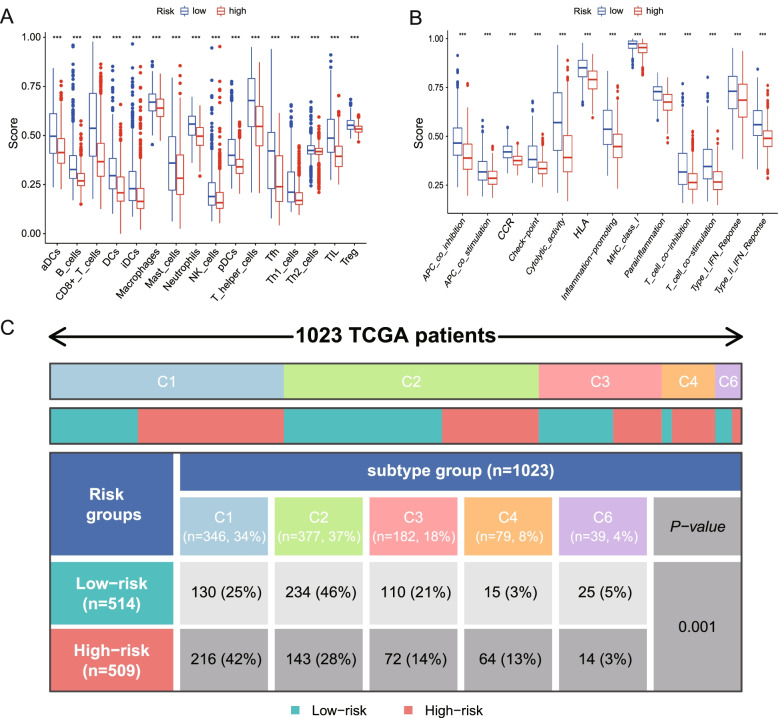
Differences of immune cells, pathways and subtypes between different risk groups. **A, B** Comparison of the enrichment scores of 16 types of immune cells and 13 immune-related pathways between low- and high-risk groups in the TCGA cohort *p* values were showed as: *p < 0.05, **p < 0.01, ***p < 0.001. **C** BRCA patients in the TCGA cohort were accordingly divided into 5 different immune subtypes. The classifications of immune subtypes were statistically different between high- and low-risk groups

SsGSEA of the TCGA cohort showed that the high-risk group all had lower levels of infiltration of immune cell infiltration than did the low-risk group (***p < 0.001, Fig. 8A). Thirteen immune pathways all showed lower activity in the high-risk group than in the low-risk group in the TCGA cohort (***p < 0.001, Fig. 8B).

BRCA patients in the TCGA cohort were divided into 5 different immune subtypes (Table S6). Most patients are C2 immune subtype in low-risk group whereas Most patients are C1 immune subtype in high-risk group. The differences were statistically different between two groups (Fig. 8C).

**Fig. 9:**
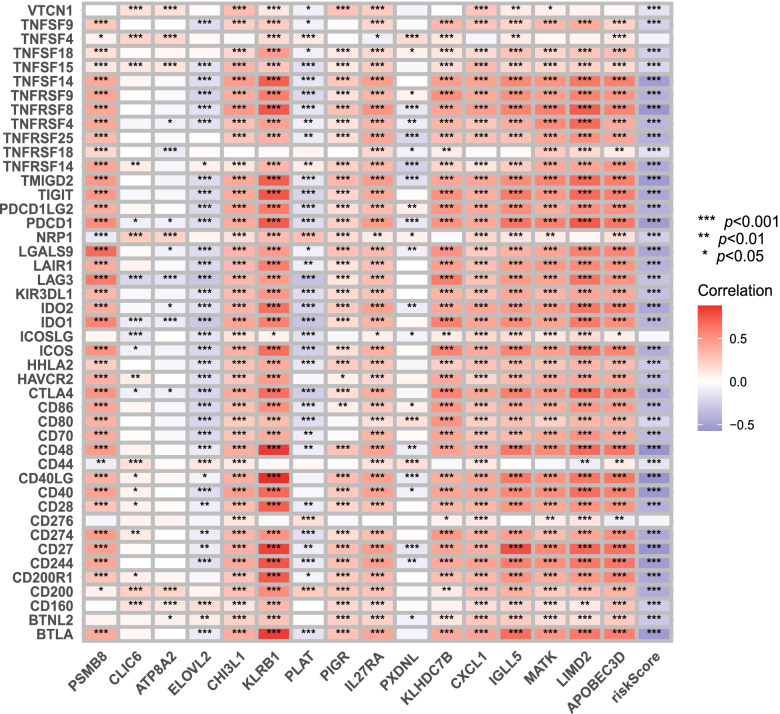
Correlations with checkpoints. The signature was negatively correlated with most checkpoints such as TIGIT and LAG3. Several genes in the signature such as CXCL1 and IL27RA were positively correlated with nearly all checkpoints

The signature was negatively correlated with most checkpoints such as TIGIT and LAG3. Several genes in the signature such as CXCL1 and IL27RA were positively correlated with nearly all checkpoints (Fig. 9).

**Fig. 10:**
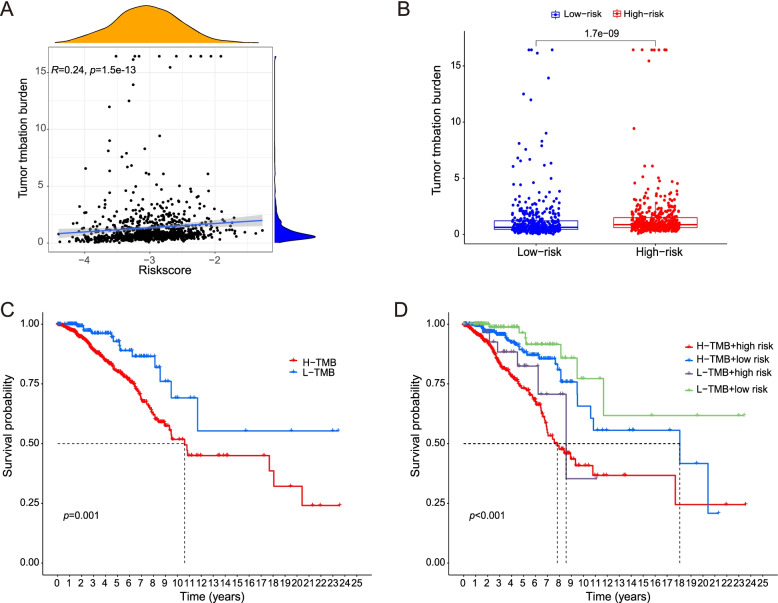
The correlation between the signature and TMB. **A** The correlation between riskscore of our signature and TMB (*R* = 0.21, *p* = 1.2e− 10). **B** The TMB is higher in high-riskscore group compared with low-riskscore group (*p* = 2.8e− 06). **C** OS of low-TMB group is better than that of high-TMB group (*p* < 0.001). **D** OS of low-TMB+ low-risk group is better than that of high-TMB+ high-risk group (*p* < 0.001)

It indicated a strong correlation between our signature and the TMB of TCGA cohort (Fig. 10A). The TMB is higher in high-risk group compared with low-risk group (Fig. 10B).

OS of low-TMB group is better than that of high-TMB group (Fig. 10C). When we mixed TMB and riskscore together, OS of low-TMB+ low-risk group is better than that of high-TMB+ high-risk group (Fig. 10D).

## Discussion

In this study, we first examined the mRNA levels of 52 currently known pyroptosis-related genes in BRCA and normal tissues and found that most of them were differentially expressed. Moreover, the two clusters produced by the consensus clustering analysis based on the DEGs did show significant prognostic value. Then, 743 DEGs in the two clusters were identified, among which 140 genes met the criteria in the univariate Cox regression analysis. A risk signature comprising 16 genes was constructed via LASSO Cox regression analysis, which was then found to perform well in the TCGA and GEO datasets. The functional analyses indicated that the DEGs between the low- and high-risk groups were strongly related to immune-related pathways. The high-risk group had universally decreased levels of infiltrating immune cells and decreased activity of immune-related pathways compared with those of the low-risk group.

Pyroptosis, a novel and recently discovered form of programmed cell death, was found to play a dual-role across different cancers in recent years. A PD-L1-mediated switch from apoptosis to pyroptosis has also been reported to facilitate tumor necrosis [26], which may promote tumor growth and impede antitumor immunity [27]. Gao et al. found that higher GSDMD expression may help tumors evade the innate immune response and indicates a poor prognosis in non-small-cell lung cancer (NSCLC) [28]. However, another study demonstrates that GSDME acts as a tumor suppressor by activating pyroptosis, enhancing antitumor immunity [29]. Pyroptosis-induced inflammation in the TME could stimulate the immune system through the activation of immune cells and immune pathways, which consequently improve the efficiency of cancer immunotherapies [30]. How pyroptosis functions in tumor tissues and influences the survival of BRCA patients remains unknown. Our study demonstrated that most pyroptosis-related genes changed with tumorigenesis and were correlated with better outcomes in breast cancer. We also generated a signature featuring 16 pyroptosis-related genes and found that it could predict OS in BRCA patients. Surprisingly, these genes were strongly related to immune cells and immune-related pathways. Based on the results of our GO and KEGG analyses, it is reasonable to speculate that pyroptosis can regulate the composition of the tumor immune microenvironment (TIME). Moreover, it also indicates a strong correlation between our signature and the TMB and the signature could to predict the TMB of breast cancer patients.

In clinical practice, monoclonal antibodies that target immune checkpoints have led to great breakthroughs in cancer therapeutics. Several PD-1/PD-L1 and CTLA-4 inhibitors have been approved for cancer treatments [31]. Due to the importance and bright prospects of immune-related treatments in cancers, more immune functions in tumorigenesis and development should be explored. The TME has diverse capacities to induce both beneficial and adverse consequences during specific stages of cancer progression and metastasis [14, 32]. Moreover, the TME is constantly evolving with tumor progression and exposure to treatment, for example, with in situ-to-invasive transition. The TME is composed of tissue-resident cells and a large proportion of recruited immune cells that can constitute up to 50% of the tumor mass in breast cancer [33]. The presence of high levels of immune cells has been associated with an improved prognosis or better response to treatment in patients with breast cancer [34]. Macrophages can also possess pro- or antitumor effects. Some previous studies indicated that tumor-associated macrophage (TAM) infiltration might increase angiogenesis, enhance tumor cell mobility and invasiveness and be associated with poor survival in breast cancer [35]. Regulatory T cells (Tregs) mainly induce immune tolerance and promote the immune escape of tumors [36]. A previous study showed that high numbers of Tregs were associated with poor survival in ovarian cancer [37]. In a metastasis model, tumor metastasis was accompanied by increased numbers of Treg cells in the primary tumors, which suggests that Treg cell recruitment to the primary tumor facilitates immune escape and tumor metastasis [38]. This means that Tregs play a deleterious role in tumor metastasis, which is inconsistent with the results of our study. Here, Tregs scored high in low-risk group. The possible reason for this discrepancy is that in advanced tumor stages, Tregs may decrease to a relatively low level as the immune function of patients is completely destroyed.

In our signature, most genes are tightly linked to the TIME in cancer. For instance, As a surface marker on several T cell subsets [39] and NK cells, killer cell lectin-like receptor subfamily B member 1 (KLRB1) encodes CD161 and reflects tumor-associated leukocytes. The expression of KLRB1 was closely associated with favorable outcomes in a pan-cancer research [40]. C-X-C motif chemokine ligand 1 (CXCL1), as a relatively specific biomarker of tumor-associated macrophages (TAMs), promotes breast cancer migration and invasion via NF-κB/SOX4 activation [41]. Chitinase 3-like protein 1 (CHI3L1), secreted by M2 macrophages, promotes the metastasis of breast cancer cells by binding interleukin-13 receptor α2 chain (IL-13Rα2) on the membranes of cancer cells [42]. Some genes contribute a lot to the riskscore, however, their roles in breast cancer were largely unstudied, for instance, Peroxidasin like (PXDNL), ATPase phospholipid transporting 8A2 (ATP8A2) and plasminogen activator, tissue type (PLAT). Maybe our research would provide some clues for subsequent studies.

Notably, immune subtype C1 makes up the largest proportion of high-risk groups whereas immune subtype C2 does of low-risk groups. C1 indicates Wound Healing immune subtype which has high expression of angiogenic genes and Th2 cell bias. C2 represents IFN-γ dominant immune subtype which has the highest M1 macrophage polarization, CD8 T cells infiltration and T cell receptor (TCR) diversity. Now we know that M1 macrophages are major players in pro-inflammatory responses while M2 macrophages are the opposite [43]. Compelling evidence point to a correlation between CD8 T cells infiltration in tumors and enhanced adaptive immune [44, 45]. It was also reported that TCR diversity positively related to overall survival in breast cancer patients [46]. Maybe all these features contribute to the relatively decreasing risk of low-risk group.

When faced with the complexity of pyroptosis and TIME, more in-depth studies are needed to guide routine clinical practice. It is ideal to identify patient populations who have a better prognosis or respond better to immunotherapeutic agents. In the last decade, next-generation sequencing (NGS) technology has emerged and allowed tremendous achievements in cancer diagnosis and analysis. With lower costs and increased, NGS has become increasingly feasible in clinical practice today [47]. When faced with huge amounts of highly complex NGS data, bioinformatics has become an indispensable method [48]. In our study, NGS data and bioinformatics methods were utilized to construct and validate a simplified pyroptosis-related signature that could not only effectively predict the prognosis of patients diagnosed with breast cancer but also indicate the degree of immune infiltration.

Currently, the mechanisms of pyroptosis have not been fully explored, especially with regard to the relationship between pyroptosis and breast cancer. Despite the drawbacks that clinical and molecular subtypes were not analysed separately and there was a lack of experimental data due to the availability of clinical specimens, we believe our studies might provide some clues for clinical decision making and subsequent studies and expand the tools available for immunotherapy.

## Conclusion

In this study, we succeeded in constructing a pyroptosis-related 16-gene signature that could predict the prognosis of breast cancer patients. The signature was also found to be closely associated with the tumor immune microenvironment and could predict the TMB of breast cancer patients.

## Supplementary Information

**Additional file 1 FigureS1.** The OS differences of 9 genes. 9 genes (GSDMA, GSDMD, GSDME, CASP1, CASP8, CASP9, NLRP1, NLRP2, NLRP7) were closely related to the occurrence of pyroptosis. Expression of the 9 genes were all positively correlated with the OS of BRCA patients (A-I).

**Additional file 2 Figure S2.** The mutational status of high- and low-risk groups. The top 20 genes most frequently mutate of high-risk group (**A**) and low-risk group (**B**). The nodes and edges of different colors represent different types of mutation.

**Additional file 3.**

**Additional file 4.**

**Additional file 5.**

**Additional file 6.**

**Additional file 7.**

**Additional file 8.**

## Data Availability

The datasets are available from TCGA (https://portal.gdc.cancer.gov/) and Gene Expression Omnibus (https://www.ncbi.nlm.nih.gov/geo/).
