# Supplementary figures and images for: Identification of a pyroptosis-related prognostic signature in breast cancer
Source: BMC Cancer. 2022 Apr 20;22:429. doi: 10.1186/s12885-022-09526-z (PMC9019977; doi:10.1186/s12885-022-09526-z)

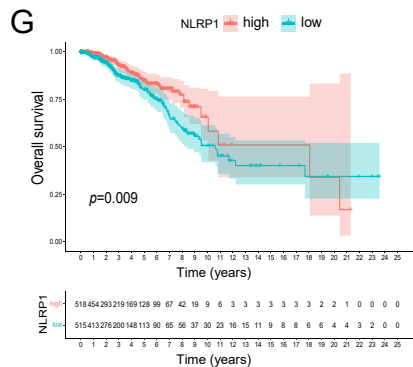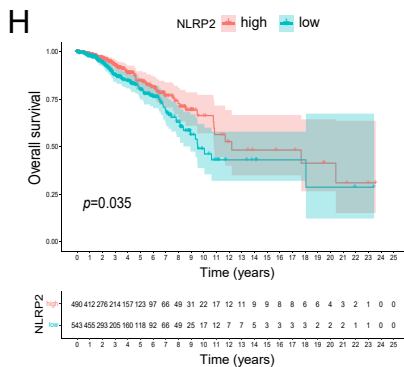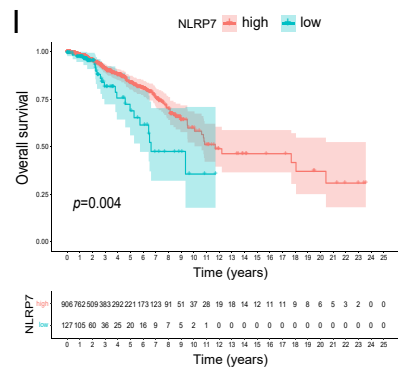

Supplement: Supplementary file 1 — Additional file 1 FigureS1. The OS differences of 9 genes. 9 genes (GSDMA, GSDMD, GSDME, CASP1, CASP8, CASP9, NLRP1, NLRP2, NLRP7) were closely related to the occurrence of pyroptosis. Expression of the 9 genes were all positively correlated with the OS of BRCA patients (A-I). [file 12885_2022_9526_MOESM1_ESM.pdf]

A

Altered in 411 (87.45%) of 470 samples.

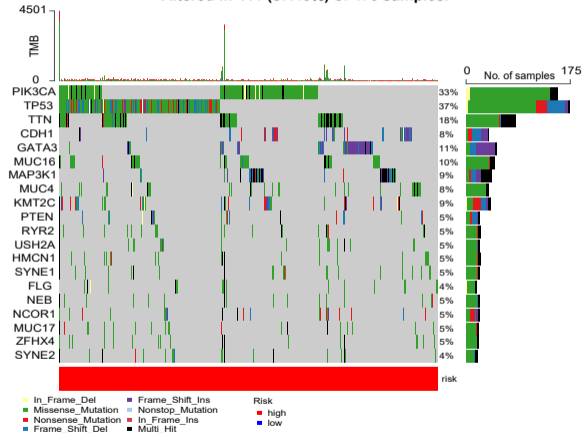

B

Altered in 387 (83.95%) of 461 samples.

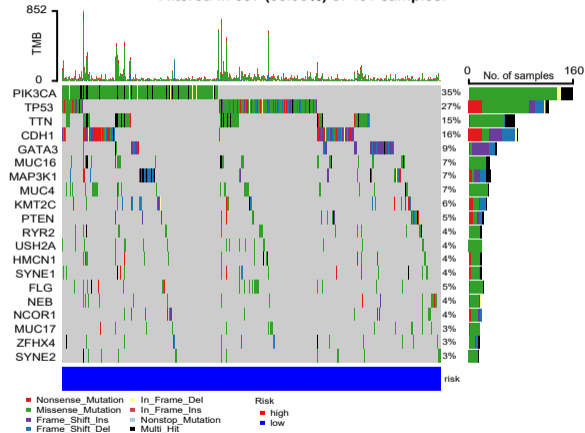

Supplement: Supplementary file 2 — Additional file 2 Figure S2. The mutational status of high- and low-risk groups. The top 20 genes most frequently mutate of high-risk group (A) and low-risk group (B). The nodes and edges of different colors represent different types of mutation. [file 12885_2022_9526_MOESM2_ESM.pdf]
