# Supplementary material for: Identification of a pyroptosis-related prognostic signature in breast cancer
Source: BMC Cancer. 2022 Apr 20;22:429. doi: 10.1186/s12885-022-09526-z (PMC9019977; doi:10.1186/s12885-022-09526-z)
Supplement: Supplementary file 3 — Additional file 3. [file 12885_2022_9526_MOESM3_ESM.docx]

**Table S1.** The 52 pyroptosis-related genes.

| **No.** | **Gene name** | **No.** | **Gene name** | **No.** | **Gene name** |
| --- | --- | --- | --- | --- | --- |
| 1 | BAK1 | 19 | IL18 | 37 | NLRP6 |
| 2 | BAX | 20 | IL1A | 38 | NLRP7 |
| 3 | CASP1 | 21 | IL1B | 39 | NOD1 |
| 4 | CASP3 | 22 | IRF1 | 40 | NOD2 |
| 5 | CASP4 | 23 | IRF2 | 41 | PJVK |
| 6 | CASP5 | 24 | TP53 | 42 | PLCG1 |
| 7 | CHMP2A | 25 | TP63 | 43 | PRKACA |
| 8 | CHMP2B | 26 | AIM2 | 44 | PYCARD |
| 9 | CHMP3 | 27 | CASP6 | 45 | SCAF11 |
| 10 | CHMP4A | 28 | CASP8 | 46 | TIRAP |
| 11 | CHMP4B | 29 | CASP9 | 47 | TNF |
| 12 | CHMP4C | 30 | GPX4 | 48 | GZMA |
| 13 | CHMP6 | 31 | GSDMA | 49 | NLRC4 |
| 14 | CHMP7 | 32 | GSDMB | 50 | NLRP1 |
| 15 | CYCS | 33 | GSDMC | 51 | NLRP2 |
| 16 | ELANE | 34 | IL6 | 52 | NLRP3 |
| 17 | GSDMD | 35 | GZMB |  |  |
| 18 | GSDME | 36 | HMGB1 |  |  |
